# Supplementary material for: Multimorbidity patterns in the working age population with the top 10% medical cost from exhaustive insurance claims data of Japan Health Insurance Association
Source: PLoS One. 2023 Sep 28;18(9):e0291554. doi: 10.1371/journal.pone.0291554 (PMC10538783; doi:10.1371/journal.pone.0291554)
Supplement: S1 Fig — The top 1%, 5%, 10%, 20%, 30% of patients accounted for 26.1%, 46.9%, 59.0%, 73.9% and 83.3% of the total annual medical costs. 1 USD = 120 JPY. (DOCX) [file pone.0291554.s002.docx]

**
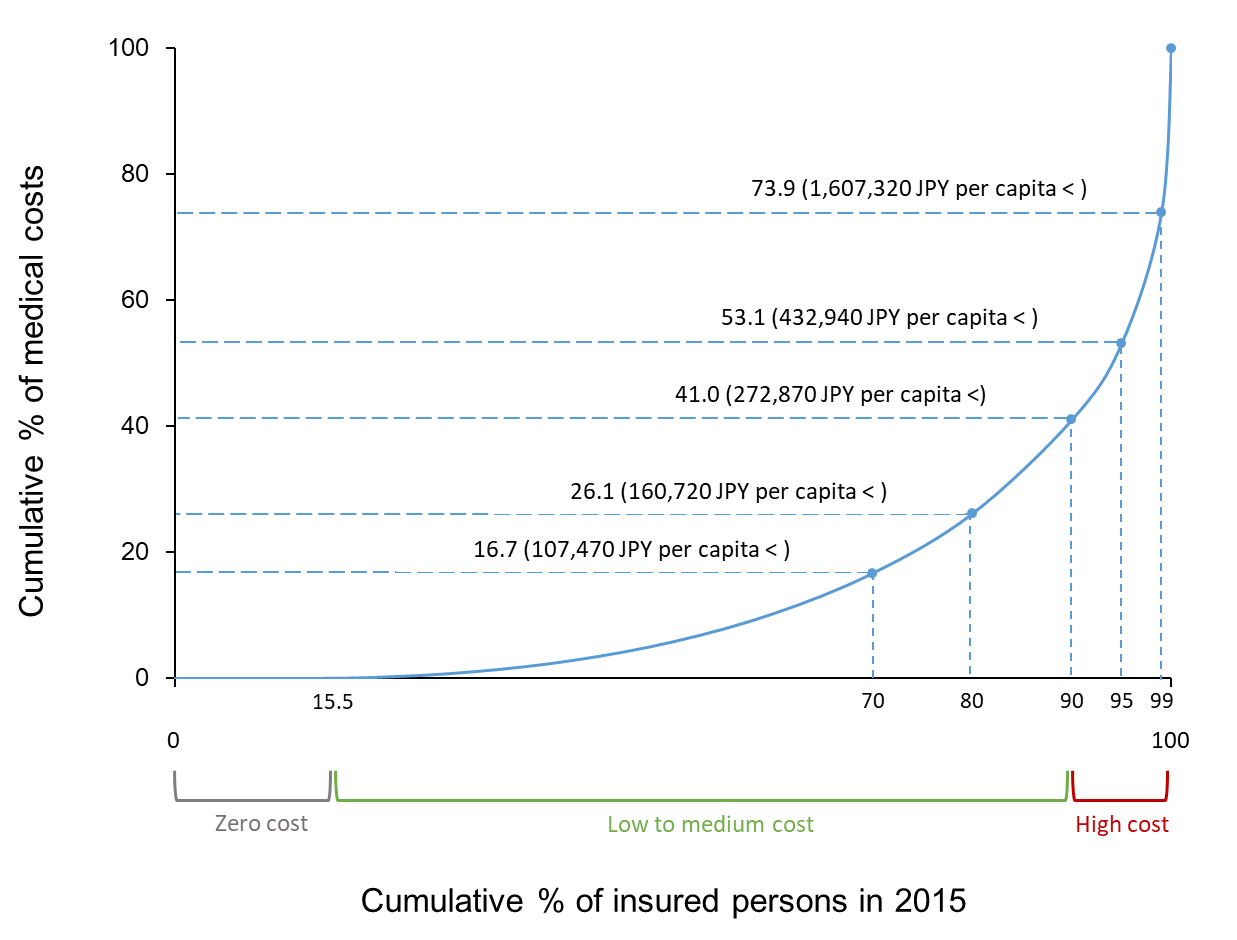
**

**S1 Fig**. **Distribution of annual medical costs in the insured population who were subscribers in 2015 (n=16,989,029).** The top 1%, 5%, 10%, 20%, 30% of patients accounted for 26.1%, 46.9%, 59.0%, 73.9% and 83.3% of the total annual medical costs, respectively.
