# Supplementary material for: Multimorbidity patterns in the working age population with the top 10% medical cost from exhaustive insurance claims data of Japan Health Insurance Association
Source: PLoS One. 2023 Sep 28;18(9):e0291554. doi: 10.1371/journal.pone.0291554 (PMC10538783; doi:10.1371/journal.pone.0291554)
Supplement: S2 Fig — BIC = Bayesian Information Criterion; AIC = Akaike Information Criterion. (DOCX) [file pone.0291554.s003.docx]

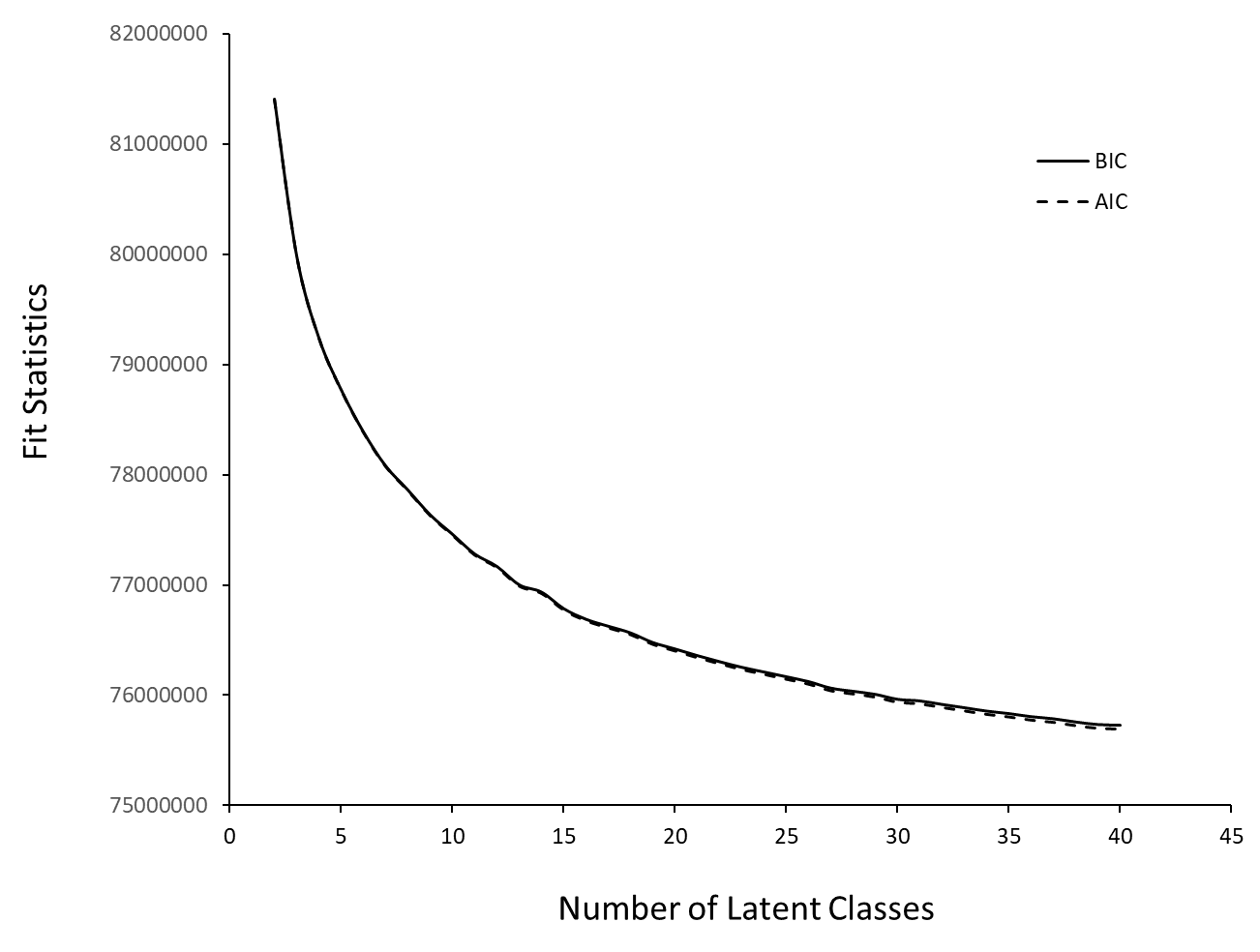


**S2 Fig**. **Model fit for the latent class analysis (BIC and AIC).** BIC = Bayesian Information Criterion; AIC = Akaike Information Criterion.
