# Supplementary figures and images for: Multimorbidity patterns in the working age population with the top 10% medical cost from exhaustive insurance claims data of Japan Health Insurance Association
Source: PLoS One. 2023 Sep 28;18(9):e0291554. doi: 10.1371/journal.pone.0291554 (PMC10538783; doi:10.1371/journal.pone.0291554)

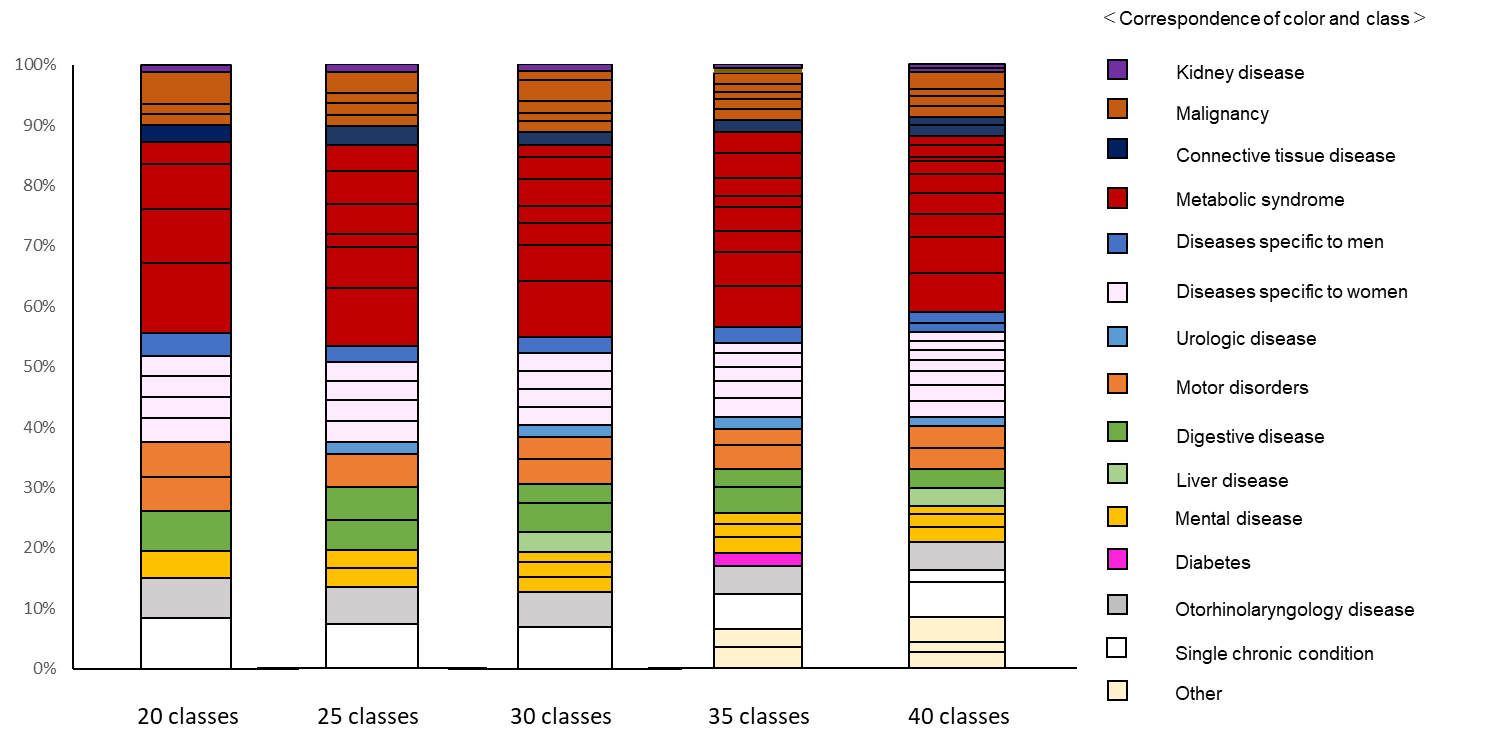


**S3 Fig**. **A 100% stacked vertical bar of the patient count in each class for the 20, 25, 30, 35, and 40 latent class models.**

Supplement: S3 Fig — (DOCX) [file pone.0291554.s004.docx]
