# Supplementary material for: Multimorbidity patterns in the working age population with the top 10% medical cost from exhaustive insurance claims data of Japan Health Insurance Association
Source: PLoS One. 2023 Sep 28;18(9):e0291554. doi: 10.1371/journal.pone.0291554 (PMC10538783; doi:10.1371/journal.pone.0291554)
Supplement: S4 Fig — Thick red represents a high probability. (DOCX) [file pone.0291554.s005.docx]

**S4 Fig**. **Heatmap for item-response probabilities of disease labels in the 30 latent class models.** Thick red represents a high probability.

Some of our multimorbidity patterns were similar to those of other studies in Asia [1]: metabolic disorders, cancer diseases, mental diseases, motor diseases, digestive diseases, pulmonary diseases, and kidney diseases. In addition, the present results also indicated that the connective tissue disease class was accompanied by typical complications, such as skin disease [2], osteoporosis [3], other locomotory diseases [4], and interstitial lung disease [5]. The connective tissue disease class had a similar 5-year mortality as the severe metabolic syndrome classes (5, 6, and 7) and several cancer classes, and requires comprehensive care with attention to complications. Although we did not include sex as a covariate in the latent class analysis, classes specific to women or men were included in this study. Patients in women-specific class 1 were the youngest of all classes and had a higher rate of perinatal disease. In Japan, medical expenses related to normal pregnancy are not covered by public medical insurance; thus, patients requiring childbirth expenses were considered to have perinatal complications and underwent an operation such as a caesarean section. The rate of inpatient cost in women in class 1 was actually the highest among all classes, which was related to a longer hospital stay after childbirth in Japan (approximately 1 week) [6]. The women-specific classes (2, 3, and 4) showed a higher frequency of uterine/ovarian cancer than the other classes. Some patients in class III also had gastrointestinal cancer, and there was a high frequency of type 2 diabetes mellitus and dyslipidemia. Moreover, the male-specific class had some features of metabolic syndrome, in addition to urological diseases.

Recent meta-analyses examined differences in medical costs by disease combinations and showed that patients with cancer and mental health conditions in the first year after cancer diagnosis incurred the highest average annual direct medical costs [7]. In contrast to the calculation of cancer medical expenses in the previous meta-analysis, not all patients in our study were in the first year of cancer diagnosis, which might have caused the medical cost for the present patients diagnosed with cancer to be lower than that for the patients in the previous study. Further studies are needed to examine the effects of cancer severity or recurrence on medical costs.

References

1. Rajoo SS, Wee ZJ, Lee PSS, Wong FY, Lee ES. A Systematic Review of the Patterns of Associative Multimorbidity in Asia. Biomed Res Int. 2021;2021: 6621785.
2. Alves F, Gonçalo M. Suspected inflammatory rheumatic diseases in patients presenting with skin rashes. Best Pract Res Clin Rheumatol. 2019;33(4): 101440.
3. Adami G, Fassio A, Rossini M, et al. Osteoporosis in Rheumatic Diseases. Int J Mol Sci. 2019;20(23).
4. Lim LSH, Feldman BM. Using Registry Data to Understand Disease Evolution in Inflammatory Myositis and Other Rheumatic Diseases. Curr Rheumatol Rep. 2019;22(1): 2.
5. Atzeni F, Gerardi MC, Barilaro G, Masala IF, Benucci M, Sarzi-Puttini P. Interstitial lung disease in systemic autoimmune rheumatic diseases: a comprehensive review. Expert Rev Clin Immunol. 2018;14(1): 69-82.
6. OECD iLibrary Health at a Glance 2017: OECD Indicators. Available from: <https://www.oecd.org/social/health-at-a-glance-19991312.htm>.
7. Tran PB, Kazibwe J, Nikolaidis GF, Linnosmaa I, Rijken M, van Olmen J. Costs of multimorbidity: a systematic review and meta-analyses. BMC Med. 2022;20: 234.
