# Supplementary material for: Multimorbidity patterns in the working age population with the top 10% medical cost from exhaustive insurance claims data of Japan Health Insurance Association
Source: PLoS One. 2023 Sep 28;18(9):e0291554. doi: 10.1371/journal.pone.0291554 (PMC10538783; doi:10.1371/journal.pone.0291554)
Supplement: Attachment — Submitted filename: Response_to_Reviewers_0822.docx [file pone.0291554.s011.docx]

Responses to Reviewer 1’s Comments

Reviewer 1

The reviewer acknowledges and appreciates the authors' efforts to incorporate our suggestions in improving the paper. Each suggestion from the reviewer has been carefully considered and addressed by the authors. There are a few additional comments to further enhance the flow and readability of the paper.

**Response:** We appreciate your valuable comments and suggestions once again. We have revised the manuscript in accordance with your comments.

**Comment 1:** Lines 41–44: As the author mentioned in the response to reviewers, only people in the working age population (under the age of 70) co-pay 30% of healthcare cost (older people are entitled to a lower co-payment).
As such, the author could consider slightly rephrasing this to: “Japan implements a universal medical care insurance system in which all citizens subscribe to healthcare insurance systems. People in the working age population (under the age of 70) receives treatment at 30% of total direct medical costs. The remaining 70% is covered by the employee’s insurance premium, of which the employee and employer each pays 50% [1].”
The sentence above has been further revised to improve phrasing and enhance clarity.

**Response:** Thank you for your suggestion. We have revised the manuscript (lines 41–43).

**Comment 2:** Line 67: Please change “chronic coexisting conditions” to “coexisting chronic conditions”

**Response:** We have revised the manuscript accordingly (line 67).

**Comment 3:** Lines 74–76: “This is because research on multimorbidity has not progressed much in Japan compared with other countries, which is related to the fact that a formal gatekeeping system has not been introduced in Japan.”
Does the author mean “multimorbidity management/care” rather than “research on multimorbidity”?
a. To improve the logical flow, please consider changing to: “The development in multimorbidity management in Japan is limited compared to other countries as there is an absence of a formal gatekeeping system in the country.”
b. Moreover, it is a bit odd that the author mentioned this point here in the introduction without further elaboration. The author may consider moving the above statement to the discussion where the author discussed the need for a more integrated patient-centered care system and the lack of primary care physicians (as in lines 360–365: “Previously, most physicians in Japan underwent postgraduate training in specialized clinical departments, leading to an emphasis on specialization and a shortage of primary care physicians. Therefore, it is necessary to increase the number of primary care physicians in the future medical policy. In recent years, the Ministry of Health, Labour and Welfare has reviewed the postgraduate training system to increase primary care physicians, and the number of primary care physicians in Japan might gradually increase in the future.”)
c. Lines 360–365 as of now seems a bit out of context. The author should consider providing more context and elaboration for this paragraph. This could be achieved by including an opening and closing statement that concisely links the point to the idea of integrated care.

**Response:** Thank you for your insightful advice. As the reviewer suggested, we agree with moving the statement ”The development in multimorbidity management in Japan is limited compared to other countries as there is an absence of a formal gatekeeping system in the country” to the discussion. In addition, we have revised the discussion keeping in mind your advice (lines 359-368): “To address the issues raised by multimorbidity, it is essential to adopt the concept of integrated care that places patients at the center of their healthcare journey. However, the development of integrated care in Japan is limited compared to other countries as there is an absence of a formal gatekeeping system in the country [43]. Previously, most physicians in Japan underwent postgraduate training in specialized clinical departments, leading to an emphasis on specialization and a shortage of primary care physicians. In recent years, the Ministry of Health, Labour and Welfare has reviewed the postgraduate training system to increase primary care physicians, and the number of primary care physicians in Japan might gradually increase in the future. By increasing the number of primary care physicians and promoting a more interconnected healthcare system, Japanese medical policy can transition towards a comprehensive and patient-focused approach to healthcare delivery.”

**Comment 4:** Line 77-78: Please change “emphasized advocating primary prevention” to “emphasized the need for primary preventions”.

**Response:** We have revised the manuscript accordingly (lines 75-76).

**Comment 5:** Line 100: Please change “formally or informally employees” to “formally or informally employed”.

**Response:** We have revised the manuscript accordingly (line 98).

**Comment 6:** Line 162-163: “The median number of 46 chronic conditions was 7 [5, 10].”
Is the author attempting to convey that "The median number of co-existing chronic conditions in an individual, out of the selected list of 46 chronic conditions, was 7 [5, 10]"?
If yes, please kindly make these changes here and in Table 2, as well as throughout the manuscript where applicable (e.g. Line 263-264).

**Response:** Yes. We have revised the sentence throughout the manuscript (lines 160-161, Table 2, Table 4, S4 Table). In relation to this revision, we have also modified the sentence “Total number of 68 diseases” to read as “Number of diagnosis codes out of the list of 68 disease labels” (lines 261-263, Table 2, Table 4, Table 5, S4 Table).

**Comment 7:** Line 180: Please revise “In preliminary analysis” to “In the preliminary analysis”.

**Response:** We have revised the manuscript accordingly (line 178).

**Comment 8:** Line 215-217: This sentence may be a bit misleading: “The kidney disease class had the smallest number of patients in all classes and showed that more than 90% of individuals had chronic kidney disease (CKD) and hypertension simultaneously.”
As the reviewer understand, more than 90% of individuals who had chronic kidney disease (CKD) also had hypertension simultaneously.
Therefore, the sentence should be revised to reflect this.

**Response:** Thank you for your suggestion. We have revised the manuscript as follows (lines 213–215): “The kidney disease class had the smallest number of patients in all classes and showed that the prevalence rates of chronic kidney disease (CKD) and hypertension were 90% and 95%, respectively.”

**Comment 8:** Line 230: Please revise “nearly half between” to “nearly half for”.

**Response:** Accordingly, we have revised the manuscript accordingly (line 228).

**Comment 9:** Line 286: Please revise to: “…multimorbidity, which was defined as both 2 or more and 3 or more chronic conditions in this study, was…”

**Response:** We have revised the manuscript accordingly (line 285).

**Comment 10:** Line 297: Please revise “recommended using” to “recommended including”.

**Response:** We have revised the manuscript accordingly (line 296).

**Comment 11:** Line 304: Please revise “The percentage of the MetS class size” to “The prevalence of the MetS class”.

**Response:** We have revised the manuscript accordingly (line 303).

**Comment 12:** Line 305: Please remove “of the population”.

**Response:** We have revised the manuscript accordingly (line 304).

**Comment 13:** Line 306: Please change “focused” to “focusing”.

**Response:** We have revised the manuscript accordingly (line 305).

**Comment 14:** Line 391-392: The author wrote “Because of this, an increase of medical cost might be affected by not only multimorbidity but the health care system…”
The author probably intend to say “Because of this, increase in healthcare service utilization might be induced by not only multimorbidity, but also the co-payment model…”

**Response:** Thank you for your correction. We have revised the manuscript accordingly (lines 394-395).

Responses to Reviewer 2’s Comments

Reviewer #2: Updated article addresses my comments and the comments of the other reviewers appropriately. I feel this article is appropriate for publication.

**Response:** We appreciate your review and feedback. Thank you for considering the article suitable for publication.
